# Dementia and Depression with Ischemic Heart Disease: A Population-Based Longitudinal Study Comparing Interventional Approaches to Medical Management

**DOI:** 10.1371/journal.pone.0017457

**Published:** 2011-02-28

**Authors:** W. Alan C. Mutch, Randall R. Fransoo, Barry I. Campbell, Dan G. Chateau, Monica Sirski, R. Keith Warrian

**Affiliations:** 1 Department of Anesthesia and Peri-operative Medicine, Health Sciences Centre, University of Manitoba, Winnipeg, Manitoba, Canada; 2 Department of Community Health Sciences, Manitoba Centre for Health Policy, University of Manitoba, Winnipeg, Manitoba, Canada; 3 Department of Psychiatry, St. Boniface General Hospital, University of Manitoba, Winnipeg, Manitoba, Canada; 4 Cardiac Sciences, Department of Surgery, St. Boniface General Hospital, University of Manitoba, Winnipeg, Manitoba, Canada; University of Bristol, United Kingdom

## Abstract

**Background:**

We compared the proportion of ischemic heart disease (IHD) patients newly diagnosed with dementia and depression across three treatment groups: percutaneous coronary intervention (PCI), coronary artery bypass grafting (CABG) and medical management alone (IHD-medical).

**Methods and Findings:**

De-identified, individual-level administrative records of health service use for the population of Manitoba, Canada (approximately 1.1 million) were examined. From April 1, 1993 to March 31, 1998, patients were identified with a diagnosis of IHD (ICD-9-CM codes). Index events of CABG or PCI were identified from April 1, 1998 to March 31, 2003. Outcomes were depression or dementia after the index event. Patients were followed forward to March 31, 2006 or until censored. Proportional hazards regression analysis was undertaken. Independent variables examined were age, sex, diabetes, hypertension and income quintile, medical management alone for IHD, or intervention by PCI or CABG. Age, sex, diabetes, and presence of hypertension were all strongly associated with the diagnosis of depression and dementia. There was no association with income quintile. Dementia was less frequent with PCI compared to medical management; (HR = 0.65; p = 0.017). CABG did not provide the same protective effect compared to medical management (HR = 0.90; p = 0.372). New diagnosis depression was more frequent with interventional approaches: PCI (n = 626; hazard ratio = 1.25; p = 0.028) and CABG (n = 1124, HR = 1.32; p = 0.0001) than non-interventional patients (n = 34,508). Subsequent CABG was nearly 16-fold higher (p<0.0001) and subsequent PCI was 22-fold higher (p<0.0001) for PCI-managed than CABG-managed patients.

**Conclusions:**

Patients managed with PCI had the lowest likelihood of dementia—only 65% of the risk for medical management alone. Both interventional approaches were associated with a higher risk of new diagnosed depression compared to medical management. Long-term myocardial revascularization was superior with CABG. These findings suggest that PCI may confer a long-term protective effect from dementia. The mechanism(s) of dementia protection requires elucidation.

## Introduction

A long-standing controversy exists as to the extent of neurological deterioration with surgical management of ischemic heart disease (IHD) [1]–[7]. Cognitive impairment, if it occurs, and progression to dementia remains poorly delineated. A confounder is that in elderly patients with IHD, early dementia may be misdiagnosed as new onset depression [8]. A further confounder is that depression may in turn affect the course of IHD [9]–[11]. Also unclear from the available literature is whether or not percutaneous coronary intervention (PCI) has a different likelihood of, or any, long-term neurological complications. In a broader sense, controversy also exists as to whether or not IHD itself causes cognitive deterioration [12]–[20]. Some recent evidence indicates that impaired cardiac output is a potential risk factor for abnormal brain aging [21], [22]. Interventions to revascularize the heart, thereby improving cardiac performance, could potentially slow cerebral deterioration over time.

Knowledge of the relationship of dementia and depression to the various management approaches for IHD would be of value to counsel cardiac patients regarding long-term survival and quality of life. The questions to consider are whether interventional approaches are more likely to precipitate cognitive decline than medical management, and if so, is PCI associated with lower risk of poor outcomes than the more invasive open-heart procedures with cardiopulmonary bypass?

In an attempt to answer these questions we have undertaken a population-based longitudinal comparison of the approach to management of IHD using data at the Manitoba Centre for Health Policy in Winnipeg, Canada. We have compared the proportion of dementia and depression in patients with previously diagnosed IHD in three groups; 1) managed by medical therapy alone; 2) managed by insertion of either bare metal or drug-eluting stents by PCI or; 3) managed by open-heart surgery with cardiopulmonary bypass.

## Methods

### Approvals

This study was approved by the Health Research Ethics Board of the University of Manitoba and the Health Information Privacy Committee of Manitoba Health. The Manitoba Centre for Health Policy works within the provisions of the Manitoba Freedom of Information and Protection of Privacy Act (FIPPA) and the Manitoba Personal Health Information Act (PHIA). Researchers do not seek individual consent to use these data for research and statistical purposes. Such use is permitted by legislation for approved research, policy formulation and/or statistical purposes.

### Data source

This study used de-identified, individual-level administrative records of health service use for the entire population of Manitoba, Canada (approx 1.1 million). The province of Manitoba provides all residents with comprehensive coverage for medical and hospital care without co-payment, as well as other health services. The research data system, housed in the repository of the Manitoba Centre for Health Policy, has been extensively validated and described elsewhere [23].

### Patient selection

**Figure 1:**
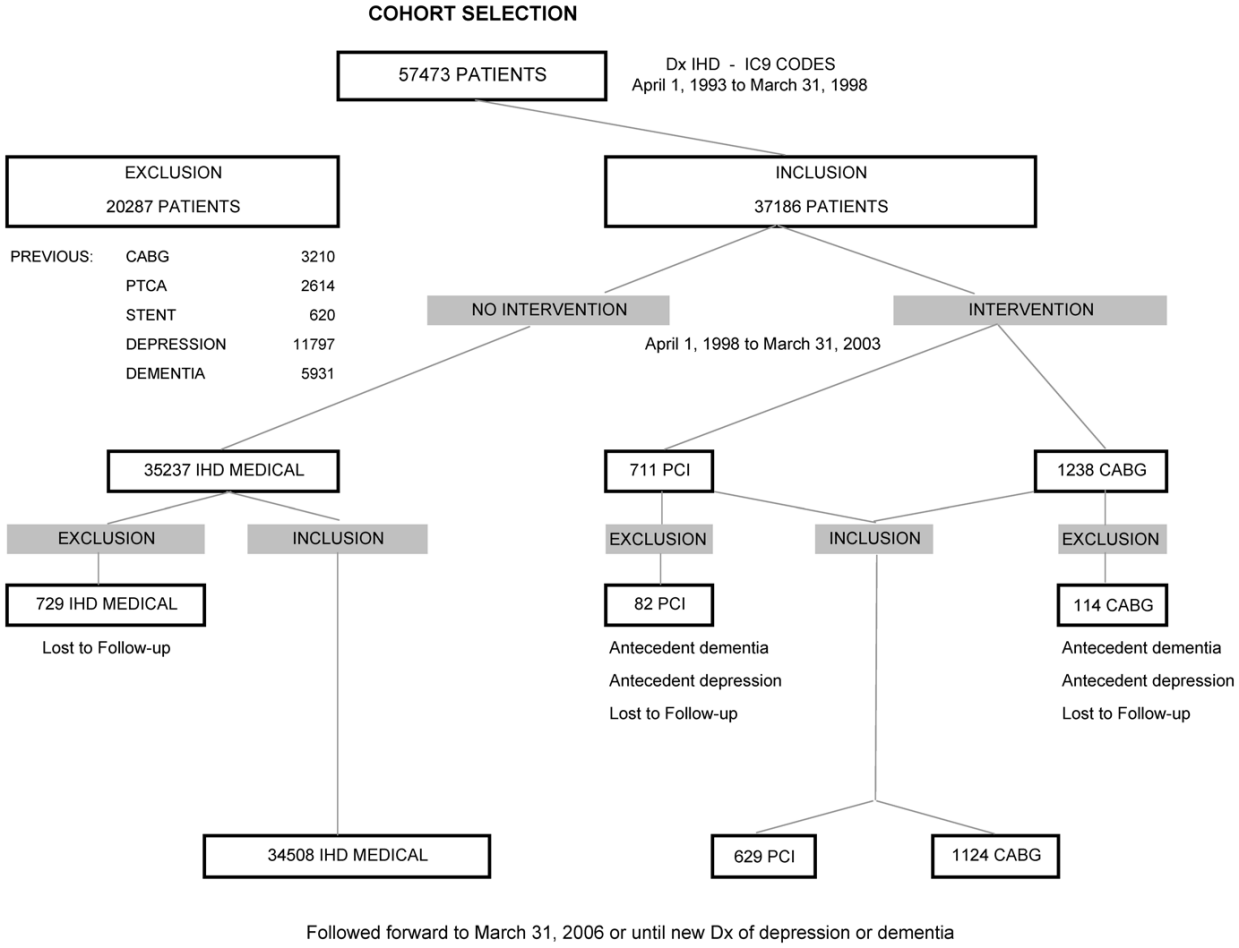
CONSORT Diagram for Cohort Selection.

Only residents age 40 years or older were included. IHD cases were identified in the five-year period from April 1, 1993 to March 31, 1998 using a definition validated by Lix et al. [24]. If a resident was hospitalized in that period and received an ICD-9-CM diagnosis between 410–414 (in any of 16 diagnosis fields), they were identified as an IHD case. Medical claims were also used to identify cases, using four overlapping two-year windows: if within any two-year period a resident had two physician visits coded with ICD-9-CM 410-414, they were identified as an IHD case. Duplicates were removed, and patients were considered IHD cases as of the first date they met either criterion. This process identified 57,473 IHD cases. We then removed patients who received any of the procedures to be examined (PCI or CABG), to eliminate the possibility that the index procedure (occurring after April 1, 1998) was not itself a subsequent procedure. We also excluded any patients with existing diagnosed dementia or depression. Dementia was defined from hospital abstract data or physician visits as any of the following ICD-9-CM diagnoses: 290.0-290.9, 291.1, 291.2, 292.82, 292.83, 294.0, 294.1, 294.8, 294.9, 331.0, 331.1, 331.7, 331.9, 797, or 799.3 [25]. Depression was diagnosed with the codes 296.2–296.8, 300.4, 309, or 311 [25]. A comprehensive report of these techniques described in “Patterns of Regional Mental Illness Disorder Diagnoses and Service Use in Manitoba: A Population-Based Study” is available [26]. See CONSORT diagram for cohort selection (Figure 1).

### Interventional cases

Records for all IHD cases were examined over the subsequent five-year period (April 1, 1998 through March 31, 2003) to track all cases receiving PCI or CABG procedures. PCI was defined as ICD-9-CM procedure codes 36.01, 36.02, 36.05, or 36.06; CABG was defined as any ICD-9-CM code 36.10-36.14 or 36.19. The first procedure in the period was taken as the ‘index’ procedure, and the case was assigned to that group; repeat/subsequent procedures were also tracked for post-hoc comparison.

### Outcomes

IHD cases comprising medical management alone, PCI or CABG cases were followed forward from the date of their procedure until March 31, 2006 (or death or loss to follow-up) to determine rates of onset of dementia or depression.

### Data Analysis

Proportional hazards regression analyses were conducted on time to diagnosis of depression and time to diagnosis of dementia, with the entire cohort of IHD cases who met the criteria described above. These models also included age, sex, diabetes and hypertension status, and income quintile (area-level average household income from the Canadian Census) as covariates. Patients were followed from the date of IHD diagnosis, until diagnosis of dementia or depression, or censoring because of death, loss to follow-up, a subsequent procedure, or the study end date (March 31, 2006). PCI status and CABG status were included as time varying covariates, such that all patients in the cohort began in the IHD group and crossed over into a treatment group at the index date of the treatment. Frequency data were compared between groups by χ^2^-tests of independence or Poisson regression. A Bonferroni correction was applied to secondary outcomes, which were considered significant at p<0.025.

## Results

The CONSORT diagram for selection criteria to the final patient cohort studied is shown in Figure 1.

The summary of patient characteristics is shown in Table 1. The largest group analyzed, by considerable margin, was the IHD-medical management group (n = 34,508) compared to PCI (n = 629) and CABG (n = 1124). The mean age was greater for the non-intervened group by 7–8 years. No difference in age was seen between the PCI or CABG groups. Male patients were more numerous in the intervened groups. Diabetes was least prevalent in the non-intervened group. Hypertension was more common in the intervened groups.

**Table 1:** Summary of Patient Characteristics.

Covariate	PCI	CABG	IHD Medical	Chi-square p-value
	n = 629	n = 1124	n = 34508	
Age (%)				
Mean Age (yr)	67.3	66.9	74.1	p<0.0001
Median Age (yr)	68	68	75	
Sex (%)				
Male	69.6	78.3	56.7	p<0.0001
Female	30.4	21.7	43.3	
Diabetes (%)				
No	72.7	65.0	83.0	p<0.0001
Yes	27.3	35.1	17.0	
Hypertension (%)				
No	38.6	33.4	56.4	p<0.0001
Yes	61.4	66.6	43.6	

The proportion of patients newly diagnosed with dementia was 4.9% for the PCI group, 7.0% for the CABG group and 12.1% for the medical management group. The mean follow-up times for the diagnosis of new dementia were 4.7, 5.2 and 4.9 years respectively (min-max; 0–8 years for all). The proportion of patients newly diagnosed with depression was 16.4% for PCI, 18.8% for CABG and 16.9% for IHD-medical management. The mean follow-up times for new depression was 4.3 years for PCI; 4.8 years for CABG and 4.6 years for IHD-medical management (min-max; 0–8 years for all).

To control for differences in covariates described above, proportional hazards regression was undertaken to allow unbiased comparisons for the likelihood of new depression and dementia for each of the studied IHD treatment modalities. Standard assumptions behind proportional hazards regression modelling were met before proceeding with the analyses.

The proportional hazards regression for covariates related to dementia is shown in Table 2. Age, female sex, presence of diabetes and hypertension, were all highly correlated with a diagnosis of dementia. The most influential variable in the model for dementia was age (χ^2^ = 1636) followed by hypertension (χ^2^ = 1012) and diabetes (χ^2^ = 233), as expected. The hazard ratio for age was 1.07, indicating advancing age was associated with proportionately greater dementia. Hypertension was associated with the greatest hazard ratio at 2.93. There was no association of income quintile with dementia. Of all patients studied, 4310 were diagnosed with new dementia for an overall proportion of 11.9% for the full cohort analyzed in the proportional hazards regression model.

**Table 2:** Proportional Hazards Regression for Covariates of Dementia.

	Hazard	95% Confidence Limits		p-value
	Ratio	Lower	Upper	
Age	1.07	1.07	1.07	<0.0001
Diabetes	1.75	1.63	1.87	<0.0001
Hypertension	2.93	2.74	3.13	<0.0001
Sex	1.10	1.04	1.17	0.017
Income	0.99	0.98	1.00	0.100

The proportional hazards regression related to new depression is shown in Table 3. Age, presence of diabetes, hypertension, female sex were all highly correlated with a diagnosis of new depression. The hazard ratio was less than 1.0 for age, indicating advancing age was associated with a proportionally lower rate of depression. There was no association of income quintile with new depression. Of all patients studied, 6215 were diagnosed with new depression for an overall proportion of 17.1% for the complete cohort analyzed in the proportional hazards regression model.

**Table 3:** Proportional Hazards Regression for Covariates of Depression.

	Hazard	95% Confidence Limits		p-value
	Ratio	Lower	Upper	
Age	0.99	0.98	0.99	<0.0001
Diabetes	1.31	1.25	1.40	<0.0001
Hypertension	2.39	2.27	2.53	<0.0001
Sex	1.62	1.54	1.71	<0.0001
Income	0.99	0.98	1.00	0.99

Table 4 shows proportional hazard ratios for dementia and depression based on IHD treatment modality. An overall test of treatment type indicated it did have a significant effect on diagnosis of dementia (χ^2^ = 6.05, p = 0.048). Pairwise comparisons revealed that intervention with PCI was associated with a lower likelihood of dementia than for medical management alone (HR = 0.65; p = 0.017). No sparing effect for dementia was seen with CABG compared to medical management (HR = 0.90; p = 0.37). PCI vs CABG showed a trend for less dementia with PCI (HR = 0.74; p = 0.15). The overall test of treatment type for diagnosis of new depression was also significant (χ^2^ = 19.7, p<0.0001). Both interventional approaches resulted in increased risk of new depression. The HR was 1.32 for CABG (p<0.0001) and 1.26 for PCI (p = 0.028). There was no significant difference between CABG and PCI interventions.

**Table 4:** Patient Outcome Hazard Ratios.

	Hazard Ratio	95% Confidence Limits Lower Upper		p-value
PCI vs IHD Medical				
Dementia	0.65	0.46	0.84	0.017
Depression	1.26	1.04	1.53	0.028
CABG vs IHD Medical				
Dementia	0.90	0.72	1.12	0.372
Depression	1.32	1.15	1.51	0.0001
PCI vs CABG				
Dementia	0.74	0.49	1.12	0.15
Depression	0.96	0.76	1.21	0.71

When comparing the two interventional groups there was a markedly different requirement for further cardiac interventions. Subsequent CABG was required in 6.3% of the patients receiving PCI as their initial intervention, nearly 16-fold greater than the 0.4% seen for patients initially revascularized with bypass surgery; (χ^2^ = 25.4; p<0.0001). Close to a third of patients with an index PCI also required re-intervention with follow-up PCI (29%) compared to 1.3% in patients who had an index CABG (a 22-fold difference; χ^2^ = 143; p<0.0001).

Death rate was 27.3% in the PCI group, 25.8% in the CABG group and 31.1% in the IHD medical group. The percent lost to follow up was 3.2% in the PCI group; 2.9% in the CABG group and 2.1% in the IHD medical group.

## Discussion

The principal new finding of this population-based longitudinal study of patients with documented IHD is that a follow-up diagnosis of dementia is less frequent with PCI as the interventional approach when compared to medical management alone. The hazard ratio for PCI as the treatment for IHD was 0.65; 95% CI: 0.46 and 0.84; p = 0.017. CABG did not confer such an advantage (HR = 0.90; p = 0.372). Both interventional approaches were associated with an increased risk of a diagnosis of new depression compared to medical management alone. That PCI as a treatment for IHD could impact on dementia risk was unexpected. Our a priori hypothesis was that patients undergoing CABG would be at greater risk of dementia than either patients managed with PCI or medical management – based on a number of studies examining cognitive dysfunction after open-heart surgery. Stated causes for increased risk of cognitive cerebral injury with open heart surgery – especially on-pump procedures – includes embolic deposition to the brain (air or particulate matter), blood pressure fluctuations, non-physiological pulsation during extracorporeal perfusion, activation of the inflammatory cascade due to blood elements contacting non-endothelialized surfaces and altered cerebral oxygenation, among others [27]–[29]. Embolic risk – although of lesser magnitude – is also present with PCI as retrograde cannulation through the aorta is required for this procedure. This lower but non-zero risk, could in theory, be hypothesized to result in a higher risk of cognitive impairment in this group compared to medically managed patients, whereas our findings indicate the opposite.

We compared our 3 groups for onset of new depression after their diagnosis of IHD because dementia is often not recognized in its earliest stages. One of our hypotheses was that physicians may misdiagnose mild dementia as depression. When patients with severe dementia are seen, a false diagnosis is less likely. Our examination of both depression and dementia was expected to capture those with certain dementia and those with more subtle changes. The reliability of using administrative data to differentiate Alzheimer's disease, vascular dementia or mixed dementia is questionable. Our definition under-estimates dementia cases to some degree, most likely the milder forms which can be more challenging to detect.

Concern could be raised about whether truly representative groups were selected to examine the proportion of dementia and depression from the total of 57,473 patients with IHD that were identified. However the proportional rate of dementia and depression in the exclusion group expressed as a percentage of the total population was 10.3% and 20.5%—similar to our results in the IHD medical group. We cede that with administrative data diagnostic criteria are more difficult to establish, and this lack of clinical correlates results in uncertainty about the appropriateness of treatment or non-treatment of patients. Also unexamined were the effects of obesity and smoking history on depression and dementia as there are no ICD-9 or -10 codes for these important covariates. As with all studies based on administrative data there is a lag time. Our data are inclusive to as recent as March 31, 2006. Current updates to clinical practice could have an impact on the findings as discussed.

The mechanism for the dementia-sparing effect of PCI can only be speculated on as this is an observational study. There is a large but controversial literature, as to whether or not neuropsychometric impairment occurs, following CABG; see references [30]–[35]. The literature relating cognitive impairment following PCI is much more sparse. However, one retrospective cohort analysis in Veterans Affairs patients comparing the emergence of Alzheimer's disease following CABG (n = 5,216) or PCI (n = 3,954) indicated an adjusted risk for dementia of 1.71 for CABG compared to patients treated with PCI; a greater sparing effect for PCI than we observed [36].

Recent work by Selnes and colleagues [5] indicates that there is no difference in cognitive performance over time in patients with IHD treated by CABG (either on-pump or off-pump) or managed medically. Our results would agree with their findings for these two groups. The study by Selnes et al. did not investigate patients undergoing PCI as a management strategy. Their work also documents that patients with IHD had lower baseline cognitive performance when compared to healthy heart subjects and had more rapid cognitive deterioration over a 72-month observation period. Other than as noted above, few studies have directly assessed neurocognitive function after PCI and compared this to either CABG or medical management alone. Small, inconclusive studies have been done by Sweet et al. [37] and Rosengart et al. [38].

That patients with coronary artery disease have an increased risk of cognitive decline is, in itself, controversial. Some studies show a relationship [3], [4], [39] while others do not [18]–[20]. Some clarification is advanced by the recently published Whitehall II study. In a large cohort of civil servants (10,308 subjects) a diagnosis of coronary heart disease was associated with decreased cognitive performance in mid-life. Cognitive function was serially assessed out to a period of ten years. When adjusted for age, education, marital status and medication for cardiovascular disease, a significant decline in reading, vocabulary and mini-mental-state-examination (MMSE) was seen when compared to those without a diagnosis of IHD. While the study highlights the impact of IHD on long-term cognitive ability, the paper does not address the impact of the management approach to IHD and its potential effects on cognition.

The Whitehall II study further highlights the importance of considering the “life-long” view of dementia. As emphasized, coronary artery disease typically manifests in mid-life with dementia occurring in later life but with a long preclinical phase.[16] As in the Whitehall II study, our observational study does not provide mechanisms to account for the identified correlation of heart disease with dementia. Potential, but unexamined advantages associated with PCI management of IHD in limiting dementia may include the following, but must be considered speculative [40]: 1) a lower occurence of congestive heart failure; 2) better control of ischemic arrhythmias; 3) a lower frequency of strokes and transient ischemic attacks; 4) more frequent carotid endarterectomy in a group of patients already pre-selected into an interventional stream, or; 5) greater exercise-initiated rehabilitation with interventional approaches. In the SYNTAX trial, patients managed with PCI had a lower rate of stroke in a 12-month follow-up period (0.6% for PCI vs. 2.2% for CABG; p = 0.003) [41].

Recent work indicates that lowered cardiac output, independent of cardiac failure, is associated with brain aging as assessed by neurocognitive testing and MRI-based neuroimaging markers predictive of preclinical Alzheimer's disease: decreased learning/memory, decreased measured whole brain and hippocampal volumes and cerebral ventricular enlargement [22]. A body of work suggests a vascular hypothesis of Alzheimer's disease in that chronic brain hypoperfusion perhaps over decades can contribute to cognitive impairment [42]–[44]. These results presuppose that lowered cardiac output affects cerebral perfusion and that cerebral autoregulation is not fully compensatory. In support of this concept is animal work where perfusion pressure was changed solely by altering pump flow to eliminate independent effects of vasopressors or vasodilators on the cerebral circulation which demonstrated attenuated autoregulation during normothermic perfusion [45]. Complete revascularization of the heart increases cardiac output following PCI [46]. One small study demonstrates superior left ventricular function in left main disease for PCI vs CABG [47]. Improved left ventricular performance, following CABG has been clearly demonstrated by some [48], [49]. More equivocal results have also been obtained following CABG in another study [50]. In this study by Diller and colleagues, left ventricular ejection velocities declined to preoperative values by 18 months, with persistent right ventricular dysfunction. Ultimately, a clear advantage for better left ventricular function and improved cardiac output following PCI compared to CABG is not truly evident from the above work.

On balance, a specific mechanism to account for the dementia-sparing effect of PCI remains elusive. It is more likely that a series of mechanisms play a part. Small net improvements of cardiac output may interplay with a lowered rate of cognitive deterioration seen with this approach as a mode of cardiac revascularization, but controversy attends these considerations. The possibility that the findings presented represent an epiphenomenon must also be considered.

It is well established that depression can impact on the nature and severity of IHD (see recent reviews) [51], [52]. There is scant literature on the question addressed here – new depression following interventional approaches to management of IHD versus medical management alone. Following CABG, readmission to hospital was correlated to pre-operative anxiety states and post-operative depression as assessed by questionnaire [53]. Amongst our 3 groups, CABG was associated with the greatest risk of depression. A lesser risk was also seen with PCI. As our study is observational, based on administrative data, we cannot comment on the severity of the depression, its mechanisms, nor its resolution with therapy. Our findings suggest that a diagnosis of new depression after the diagnosis of IHD is common; greater than 17% for the full cohort studied. Treatment of depression in patients with IHD may provide long-term improved quality of life [54], [55]. Our finding of an inverse relationship between advancing age and the occurrence of depression has previously been documented for the Province of Manitoba in the MCHP database, by Martens et al. [26].

One of the strengths of our study is its long follow-up time. We have followed patients for a minimum of three years and to a maximum of 8 years after the diagnosis of IHD to identify dementia and depression.

Limiting other end-organ damage with interventional approaches to IHD is now especially important as new evidence is suggesting that CABG may be superior to stent insertion for managing IHD [41], [56]. Our data, in part, further address this issue. The quality of coronary revascularization was noticeably better with CABG compared to PCI, with a 16-fold lower rate of subsequent open-heart surgery following an initial bypass procedure and a 22-fold lower rate of subsequent PCI. Other recent work has also indicated that CABG was superior to PCI for myocardial revascularization in a non-inferiority study [41]. Our study suggests ever greater superiority for CABG over PCI. The difference, in part, relates to our follow-up time of up to 8 years vs the SYNTAX trial follow-up of 12 months. We cannot comment on whether or not some of the follow-up PCI interventions may have been deliberately planned as staged procedures. An argument advanced is that PCI should be considered for more frail patients; however, the recent work by Hannan et al. [56] indicates that their CABG patients had greater cardiac and other end-organ disease pre-operatively.

We acknowledge that our finding regarding the dementia-sparing effect of PCI is controversial and provocative. Follow-up studies that are able to control for disease and outcome severity, especially prospective ones to confirm or refute these findings are required. We show that PCI as an interventional approach decreases the likelihood of dementia in patients with documented IHD. If our results are confirmed the means chosen to manage IHD may influence the frequency of dementia and as a consequence the quality of life in long-term survivors in this patient population. Our study suggests that there are both cardiac and cerebral benefits to interventional management of IHD. The optimal approach to manage patients with IHD may now need to balance the approach for long-term revascularization of the heart to the differing long-term attenuation of dementia that occurs with a chosen interventional approach. This study suggests that prospective comparative effectiveness research [57] is warranted to answer the issues raised.
